# Comparison of Olfactory Function before and After Endoscopic Sinus Surgery

**Published:** 2018-01

**Authors:** Seyed Javad Seyed Toutounchi, Mohamad Yazdchi, Rana Asgari, Negisa Seyed Toutounchi

**Affiliations:** 1 *Department of Otorhinolaryngology, School of Medicine, University of Medical Sciences, Tabriz, Iran.*; 2 *Department of Neurology, School of Medicine, University of Medical Sciences, Tabriz, Iran*; 3 *Department of Pharmacy, University of Medical Sciences, Tabriz, Iran.*

**Keywords:** Nasal polyp, Nasal endoscopic surgery, Olfaction, Sinusitis

## Abstract

**Introduction:**

Olfactory loss in patients with chronic rhinosinusitis has been measured by different methods. However, the results have been variable, and it is not clear whether endoscopic sinus surgery significantly improves olfactory function. This study was performed to evaluate the influence of endoscopic sinus surgery on olfactory function in patients with chronic rhinosinusitis.

**Materials and Methods:**

In this prospective analytic study, 73 patients (mean age, 39.63±12.94 years) with a diagnosis of polyps and sinusitis during 2011 were studied. The olfaction test was performed with three solutions; one with no odor (water) and two with phenylethyl alcohol (50% and 90% dilution, respectively). The patients’ olfaction state were graded as no olfaction, or low, moderate or good olfaction before and 1 and 3 months after surgery, and was given scores between 0 and 3 and evaluated quantitatively.

**Results:**

Right-side olfaction was improved in 68.5% and left side in 67.1% of patients. Mean olfaction score on the right and left side was significantly improved after surgery in comparison with basic scores (before and after on the right side: 0.95±0.88 and 2.02±1.04; before and after on the left side: 1.02±0.84 and 2.00±1.21; both P<0.001). Improvement after surgery in cases with left- and right-side anosmia was 66.7% and 61.9%, in low olfaction was 82.3% and 72.7% and in moderate olfaction was 66.7% and 80%.

**Conclusion:**

In patients with rhinosinusitis, endoscopic sinus surgery has considerable effect in improving olfactory function.

## Introduction

The olfactory sense has a protective role against environmental risks (such as spoiled food, gas leaks and smoke) and helps with gestation; thus it is an essential factor in determining quality of life (1). Patients with olfactory dysfunction usually face problems in cooking, changes in behavior, reduced appetite, and awareness of self-hygiene (2).

A normal sense of smell is consistent with important physiological pathways. The smell stimulates the neuronal receptors of the olfactory epithelium in the nasal upper posterior septum, olfactory cleft and upper parts of the middle and anterior cornea, and triggers the neural cascade via the olfactory bulb, and consequently stimulates the olfactory cortex. Any disruption in this pathway would lead to loss of the sense of smell. There are many pathologic causes for olfactory dysfunction, but the major causes are viral infections, sinonasal diseases (such as chronic rhinosinusitis and nasal polyp) and traumatic injuries (3-5).

The main cause of olfactory disorders in patients is nasal polyps and airway obstruction which blocks pathway of the air contacting the smell-sensitive receptors. Another less probable cause is injury to the olfactory system due to microbial toxins. Hence, removing the polyps and discharging the sinuses and controlling the infections usually improves the sense of smell (6,7).The main question that patients with olfactory dysfunction usually ask before surgery and the treatment of polyps is about the recovery of the sense of smell, and the answer to this question is actually difficult. Although there have been studies reporting improvement in olfactory function after surgery (8-11), the correlation between the rate of recovery and age, gender, duration of the disease, previous medications, other accompanied disorders and involved sinuses, has not yet been defined properly. In the present study, all parameters affecting the rate of olfactory recovery after surgery were evaluated including age, gender, disease duration, olfactory dysfunction duration, type and duration of previous medications, other accompanied disorders, involved sinuses, and degree of olfactory dysfunction.

*Review of articles*

Olfactory dysfunction is usually the chief complaint of the patients with nasal polyposis and rhinosinusitis. It causes even more problems in patients with specific jobs that demand a keen sense of smell.

Smith et al. evaluated 320 patients from 2004 to 2008 with rhinosinusitis in whom olfaction improved after endoscopic nasal surgery, and assessed the influencing factors. Approximately 15.8% of patients with allergic rhinosinusitis and 12.2% of patients with chronic sinusitis had a remarkable recovery in all aspects of quality of life. Patients undergoing their first surgery had a better chance of recovery than those with previous surgical history (9).

Moreover, Litvack et al. evaluated the effect of endoscopic rhinosinus surgery on improvement in olfactory sense in patients with chronic rhinosinusitis in the long term. In this study, 111 patients were under observation for 6 to12 months. The rate of sex-dependant olfactory disorder incidence before surgery was 67.5%. There was no significant improvement in hyposmic patients after surgery; however, anosmic patients showed a remarkable improvement after endoscopic nasal surgery, which was permanent for next 12 months (10).

In another study by Litvack et al., the clinical characteristics of olfactory function in 367 patients with chronic rhinosinusitis were evaluated. The results showed that 64% of male and female patients between 18 and 64 years of age had olfactory dysfunction. Lojestic regression analysis introduced age, smoking, nasal polyposis and asthma as the most important factors in the incidence of olfaction problems in patients (11).

In a prospective study, Hummel and Pade investigated the determinant factors of olfaction after nasal surgery. Thus, 775 patients in the age range of 10–81 years old were studied. After sinusoidal surgery, olfaction recovery was seen in 23% of patients. No significant change was observed in 68% of patients, and olfactory function was worsened in 9% of patients. Age and gender had no considerable effect on surgery outcome in terms of olfactory function (12).

In a study by Perry and Koutakis, 178 patients were evaluated in order to determine the effect of endoscopic surgery of the sinuses on subjective olfactory dysfunction in patients with chronic rhinosinusitis. The mean score of olfactory dysfunction before the surgery was 4.9, and reduced to 0.9 a year after the surgery. The higher score on the computed tomography (CT) scan and accordingly higher score of olfactory dysfunction were followed by a higher rate of recovery after surgery. Patients with asthma and polyposis had a higher rate of subjective olfactory dysfunction. All of the groups showed significant improvement after 1 year (13).

In a study by Delank and Stoll, 115 patients were studied, 58% of whom had an olfactory complaint before surgery. Olfactory tests revealed hyposmia in 52% and anosmia in 31% of the patients. Olfactory recovery after surgery was observed in 70% of cases. Improvement to the normal olfaction was seen in 25% of hyposmic and only 5% of anosmic patients and in 8% of patients the olfaction condition was worsened after endoscopic sinusoidal surgery (14).

Minovi et al. studied 64 patients between the ages of 22 and 67 years. Age, asthma incidence and previous surgical interventions had no significant effect on surgery outcome, in terms of olfaction. Olfactory improvement after nasal surgery could last for 6 months on average (15).

In a study by Schriver et al. the effect of nasal surgery on olfactory function was evaluated for 12 months. In total, 157 cases were followed up for 3.5 months and 52 cases were followed up for 12 months. Olfactory function was remarkably improved 3.5 months after rhinosinusoidal surgery, while the olfactory improvement within 12 months was not noticeable. However, olfactory recovery after 3.5 and 12 months was reported in 19% and 17% of patients, respectively (16).

Moreover, in a study by Jiang et al., the effects of endoscopic rhinosinusoidal surgery on olfactory outcomes in patients with chronic rhinosinusitis were evaluated. In this research, the rate of patients with olfactory dysfunction before and after surgery was reported as 74.3% and 68.6%, respectively. In patients with severe rhinosinusitis, without considering the evaluation method, surgery had minimal effect on the sense of smell. This study determined that endoscopic rhinosinusoidal surgery has no effect on olfactory improvement (17).

Another study by Jiang et al. aimed to determine the predicting factors of olfactory recovery rate after endoscopic rhinosinusoidal surgery. The results showed no considerable correlation between nasal obstruction degrees, severity of chronic rhinosinusitis, nasal polyposis or allergic conditions (18).

In 2010, Solar et al. studied the effect of histologic markers on olfactory outcome after endoscopic rhinosinusoidal surgery. Results of this study showed that olfactory dysfunction is directly related to the level of tissue eosinophils and thickness of basal membrane. Thus, it can be suggested that most of patients with chronic rhinosinusitis and olfactory dysfunction, suffer from permanent neuroepithelial degeneration (19).

Additionally, Shin et al. evaluated olfactory function after endoscopic rhinosinusoidal surgery. The olfactory cleft condition obviously affected the olfaction score before surgery. Within 2 months after surgery, subjective symptoms and objective olfactory threshold were improved in 96% and 68% of patients, respectively. There was no relationship between subjective symptoms and olfactory improvement (20).

## Materials and Methods

In this prospective longitudinal study, 73 patients referring to the ear, nose and throat (ENT) section of Imam Reza Hospital in Tabriz with nasal and sinus polyp diagnosis who were hospitalized and underwent endoscopic nasal and sinus surgery in 2011–2012 were studied. Patients with olfactory dysfunction who had a post-surgery history, those who had olfactory problems before nasal polyps, diabetic or asthmatic patients, or patients with any other systemic disease interfering with the olfactory function, and users of autotoxin drugs were excluded from this study.

All personal information including age, gender and disease characteristics such as symptoms of nasal congestion, nasal and post-pharyngeal discharge, headache, coughing, olfactory dysfunction and its duration, other accompanied disorders, drug administration, previous surgery history, radiographic findings, and physical examination findings from nasal and sinuses were listed for each patient. The olfactory function of both sides of the nose before and 1 and 3 months after surgery were scored from 0 to 3, as none, low, moderate, or normal olfaction. Patients’ olfaction was tested using an odorless substance (water) or phenylethyl alcohol at concentrations of 50% and 90%. The solutions were placed in separate bottles numbered as 1, 2 and 3, and the examiner and patients were unaware of the contents of the bottles. In case of suspicion of malingering, isotope scanning was used; however, due to the high costs of this test, there was no possibility of testing all patients with isotope scanning. All patients signed written consent before entering the study after the whole research program was explained to them.

All data were statistically analyzed using SPSS16, and descriptive analytical methods (frequency, percentage, mean, and standard division) were used. A paired samples test was used for comparing quantitative parameters, and P<0.05 was considered significant.

## Results

**Fig 1:**
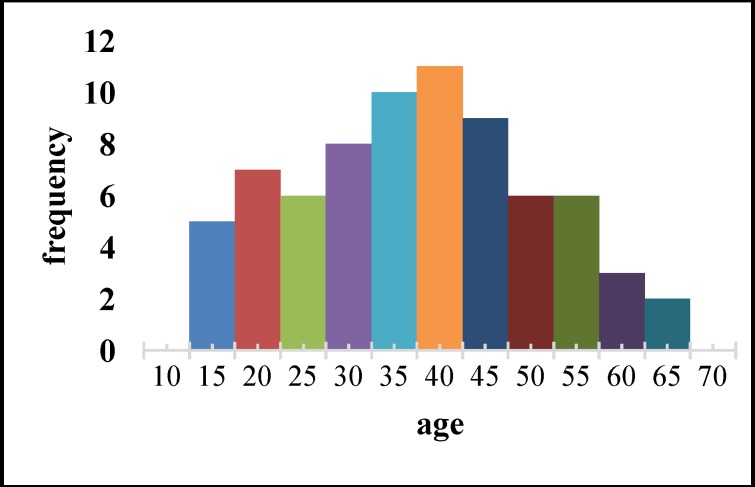
Age range of patients studied

The age range of the studied patients is shown in Figure 1. The median age was 40 years old. The youngest and oldest patients were 18 and 68 years old, respectively. Forty-one patients (56.2%) were male and 32 (43.8%) were female. The average disease duration was 2.95±1.44 years, with a median of 3 years. The shortest and the longest period of disease were 1 and 6 years, respectively. Twenty-three patients (31.5%) had a history of smoking.

*Clinical symptoms*

Clinical symptoms included nasal congestion in 100% of patients, post-pharyngeal secretions in 91.8% of patients, facial pain in 57 patients (78.1%), rhinorrhea in 41 patients (56.2%) and cough in 40 patients (54.8%). None of the patients had any intubation or history of intensive care unit (ICU) admission. Thirty-three patients (42.2%) had a history of general anesthesia and two patients (2.7%) had a previous nasal trauma. None of the patients had any facial trauma, radiotherapy or history of chemotherapy.

*Findings from nasal endoscopy*

Nasal polyps were observed in six patients (8.2%) on the right, and in six patients (8.25) on the left side, and 61 patients (83.6%) had bilateral polyps. Cornea enlargement, purulent nasal discharge, post-pharyngeal discharge and septal deviation were observed in 61 (83.6%), 17 (23.3%), 14 (19.2%) and 49 cases (67.1%), respectively.

*Findings from CT scan*

The findings from the CT scans of the patients are listed in Table 1. There was a nasal mass in 53 cases (72.6%). It can be seen from the table that the least involved sinus was the sphenoid sinus.

**Table 1:** CT scan findings

	**Number**	**Right**	**Left**	**Bilateral**
**Maxillary sinusitis**	73	6 (8.2%)	8 (11%)	59 (80.8%)
**Ethmoiditis**	73	6 (8.2%)	6 (8.2%)	61 (83.6%)
**Frontal sinusitis**	70	15(21.4%)	16 (22.9%)	39 (55.7%)
**Sphenoid sinusitis**	59	7 (11.9%)	15 (25.4%)	37 (62.7%)

*Olfactory situation before and after surgery*

**Fig 2:**
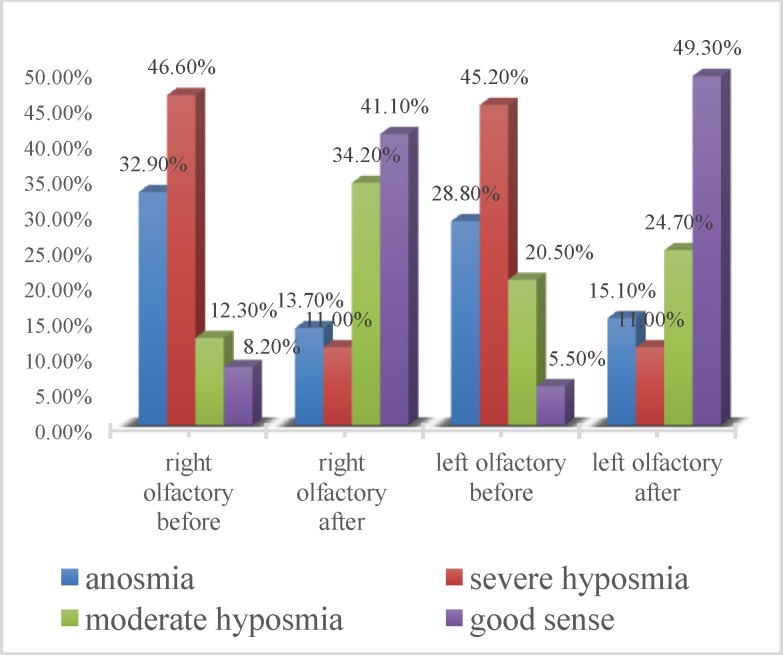
Olfactory condition in both nasal cavities before and after surgery

Figure 2 shows the olfactory condition in both nasal cavities before and after surgery. As shown in the figure, there was an increase in cases of good olfaction and mild hyposmia in both nasal cavities after surgery, and anosmia was almost cured.

*Evaluation of olfactory function*

Anosmia: In 66.7% of 24 patients with right-side anosmia, olfaction improvement was observed as follows: three cases (12.5%) revealed hyposmia, seven cases (29.2%) revealed mild olfaction and six patients (25%) showed good olfaction. Likewise, 61.9% of 21 cases of left-side anosmia had an olfaction improvement to hyposmia in two cases (9.5%), mild olfaction in five cases (23.8%) and good olfaction in six cases (28.6%).

Hyposmia: from 34 cases of right-side hyposmia, no significant changes in olfaction was seen in 14.37% of patients, and in one case the olfactory condition was worsened to anosmia. Meanwhile, 82.3% of patients revealed good progression to mild olfaction in 15 cases (44.1%) and good olfaction in 13 cases (38.2%). Likewise, in 33 patients with left-side anosmia the recovery rate was 72.7% including 10 cases (30.3%) of mild olfaction and 14 cases (42.4%) of good olfaction. However, in three cases (9.1%), anosmia happened after surgery. Mild olfaction: from nine cases of mild olfaction on the right side, six cases (66.7%) had an improvement to normal olfaction and from 15 cases of mild olfaction on the left side, 12 cases revealed complete recovery of olfactory function. Good olfaction: from six cases of normal olfaction of the right side, complete anosmia occurred in one patient after surgery, while all four patients with normal olfaction on the left side remained unchanged after surgery.

Overall, right-side olfaction was improved in 50 patients (68.5%) and left side olfaction was improved in 49 cases (67.1%).

The olfactory condition of both sides of the nose before and after surgery were evaluated and classified as none, hypo, mild and good olfaction and scored from 0 to 3. The results were analyzed quantitatively as followed: 1) The average of olfactory score on the right side before surgery was 0.95±0.88 and after surgery was 2.02±0.04 (P<0.001); 2) On the left side, the average olfactory score before surgery was 1.02±0.84 and after surgery was 2.00±1.21 (P<0.001). Considering the recovery of both or one side olfaction as complete recovery, 55 cases (75.3%) revealed complete recovery. The results of both the recovered and none recovered group is listed in Table 2. No significant difference between groups was observed; thus, it is not possible to determine the factors affecting the outcome of endoscopic surgery.

**Table 2:** Results of olfaction changes in the recovered and non-recovered group

**Characteristic**		**Recovered**	**Non-recovered**	**P-value**
Age		38/20±13/44	44/00±10/42	0/09
Sex	Male	12 (66/7%)	29 (52/7%)	0/41
	Female	5 (33/3%)	26 (74/3%)	
Duration of disease		3/44±1/58	2/80±1/37	0/1
Smoking		7 (41/2%)	16 (29/1%)	0/38

## Discussion

In the present study, the role of endoscopic sinus and polyp surgery in the improvement of olfaction in patients with chronic rhinosinusitis was investigated. In this study, the rate of anosmic patients on the right and left side were 32.9% and 28.8%, respectively, and 8.2% and 5.5% of patients had normal olfaction on the right and left side, respectively. The rate of olfactory improvement on the right side was 68.5% and on the left side was 67.1%. Although anosmia occurred after surgery in two cases (2.74%), the overall olfactory scores were significantly increased in both sides after surgery.

Results from the study of Ennhage et al. also confirmed that nasal endoscopic surgery significantly improves all olfactory parameters, including subjective and objective tests (21). In a similar study by Delank and Stoll, the rate of olfactory improvement after surgery was 70% (14). Litvack et al. had reported olfactory dysfunction in 61–83% of patients with chronic rhinosinusitis, while the highest level of olfactory dysfunction occurred in patients with nasal polyposis (8). 

Additionally, in an investigation by Pade and Hammel, the rate of recovery of olfaction after surgery was 23%, while in 68% of patients no significant changes were observed and in 9% of patients, the olfactory function was declined (12). Likewise, in a study by Perry and Jountakis, a significant decrease in the subjective olfaction of patients was reported 1 year after nasal surgery (13).

In contrast, in a study by Jiang et al., it was observed that endoscopic surgery of the sinus and nose in patients with chronic rhinosinusitis had no considerable effect on olfactory function. In this study 74.3% and 68.6% of patients had olfactory dysfunction before and after surgery, respectively (17).

The variation in results of the studies mentioned above could be due to the sample selection and the number and severity of the problems. In the present study, the rates of recovery of the left and right-side olfactory function in anosmic patients were 66.7% and 61.9% and in hyposmic patients were 82.35% and 72.7% and in moderate olfaction were 66.7% and 80%, respectively. Complete recovery to normal olfaction of the right and left side in anosmic patients were 25% and 28.6%, respectively, while in hyposmic patients were 38.2% and 42.4% and in patients with moderate olfaction were 66.7% and 80% in the right and left side, respectively. It can be deduced that the lower the severity of olfactory dysfunction, the higher the rate of recovery to normal olfaction.

In a study by Delank and Stoll, normal olfaction was achieved in 25% of hyposmic patients and only 5% of anosmic patients after surgery (14); despite the of low rate of recovery, a higher rate of improvement was seen in less severe problems. However, in the study by Litvack et al., the olfactory improvement after endoscopic surgery in anosmic patients was higher than in hyposmic patients (10).

Litvack et al. reported olfactory dysfunction in 61–83% of patients with chronic rhinosinusitis, as the higher incidence of olfactory dysfunction was observed in patients with nasal polyposis. It was observed that patients with olfactory dysfunction significantly had worse endoscopic score (normal olfaction: 4.16±3.97, hyposmic: 6.26±4.21, anosmic: 9.61±4.48) and worse CT scan score (normal olfaction: 9.11±5.4, hyposmic: 11.16±5.96, anosmic: 17.62±5.37). In this study, the severity of olfaction reduction had a direct relationship with the severity of sinusitis in CT scan and nasal endoscopic examination. The olfactory function had no relation to quality of life (8).

In a meta-analysis into the use of pre- and postsurgical olfactory outcomes to assess the impact of endoscopic sinus surgery on chronic rhinosinusitis-related olfactory impairment, Kohli et al. reported that endoscopic sinus surgery improves nearly all subjective and objective measures of olfaction in chronic rhinosinusitis patients. Patients with nasal polyposis or preoperative olfactory dysfunction improved to a greater degree (22).

Studies showed that basal olfactory condition and nasal polyposis are closely related to the rate of olfaction improvement after surgery (10). Moreover, Litvack et al. observed that age, smoking, nasal polyposis and asthma are important factors in generating olfactory problems. Gender had no effect in this case (11).

In the present study, age and gender had no significant effect on the recovery and none of the investigated factors were able to anticipate the outcome of surgery. Similarly, Pade and Hummel reported that age and gender had no significant effect on surgery outcome on olfaction (12). Also, Minovi et al. observed that age, asthma and previous surgical interventions had no considerable effect on the results of the surgery (15). However, Alt et al. showed that recalcitrant disease and aspirin intolerance were strongly predictive of poorer olfactory function (23). We excluded patients with high risk of olfactory dysfunction from our study.

Smoking did not show such a correlation with symptom alteration during follow-up (P=0.338). Minor recurrence (general edema of the mucosa or minor polyposis) was found in 32 patients (51.6%). The recurrence rate was related to associated diseases and the severity of symptoms at presentation in the study by Bakhshaee et al. (24). In another study by Bakhshaee et al., the patients’ most common symptoms in chronic rhinosinusitis were nasal discharge (95%), blockage (94%), smell disorders (63%), cough (45%), halitosis (41%), lethargy (37%), and aural fullness (36%) (25). This was similar to our study with nasal congestion in 100% of patients, post-pharyngeal secretions in 91.8% of patients, facial pain in 57 patients (78.1%), rhinorrhea in 41 patients (56.2%) and cough in 40 patients (54.8%).

## Conclusion

Endoscopic surgery of the nose and sinuses in patients with rhinosinusitis has a considerable positive effect on olfactory function, and significantly improves the sense of smell.

*Suggestions*

Endoscopic surgery of the nose and sinuses especially in patients with chronic rhinosinusitis is recommended for improving the olfaction in patients. In this study, no significant correlation between different parameters and olfactory recovery was seen. Further investigations with larger number of patients would provide better and more accurate results.

## References

[1] Neuland C, Bitter T, Marschner H, Gudziol H, Guntinas‐Lichius O (2011). Health‐related and specific olfaction‐related quality of life in patients with chronic functional anosmia or severe hyposmia. The Laryngoscope.

[2] Temmel AF, Quint C, Schickinger-Fischer B, Klimek L, Stoller E, Hummel T (2002). Characteristics of olfactory disorders in relation to major causes of olfactory loss. Arch Otolaryngol Head Neck Surg.

[3] Vent J, Robinson A M, Gentry‐Nielsen M J, Conley DB, Hallworth R, Leopold D A (2004). Pathology of the olfactory epithelium: smoking and ethanol exposure. The Laryngoscope.

[4] Holbrook EH, Leopold DA (2006). An updated review of clinical olfaction. Curr Opin Otolaryngol Head Neck Surg.

[5] Brämerson A, Nordin S, Bende M (2007). Clinical experience with patients with olfactory complaints, and their quality of life. Acta Otolaryngol.

[6] Stevens MH (2001). Steroid‐Dependent Anosmia. The Laryngoscope.

[7] Kern RC (2000). Candidate's Thesis: Chronic Sinusitis and Anosmia: Pathologic Changes in the Olfactory Mucosa. The Laryngoscope.

[8] Litvack JR, Mace JC, Smith TL (2009). Olfactory function and disease severity in chronic rhinosinusitis. Am J Rhinol Allergy.

[9] Smith TL, Litvack JR, Hwang PH, Loehrl TA, Mace JC, Fong KJ (2010). Determinants of outcomes of sinus surgery: a multi-institutional prospective cohort study. Otolaryngol Head Neck Surg.

[10] Litvack JR, Mace J, Smith TL (2009). Does olfactory function improve after endoscopic sinus surgery?. Otolaryngol Head Neck Surg.

[11] Litvack JR, Fong K, Mace J, James KE, Smith TL (2008). Predictors of olfactory dysfunction in patients with chronic rhinosinusitis. The Laryngoscope.

[12] Pade J, Hummel T (2008). Olfactory function following nasal surgery. The Laryngoscope.

[13] Perry BF, Kountakis SE (2003). Subjective improvement of olfactory function after endoscopic sinus surgery for chronic rhinosinusitis. Am J Otolaryngol.

[14] Delank K, Stoll W (1998). Olfactory function after functional endoscopic sinus surgery for chronic sinusitis. Rhinology.

[15] Minovi A, Hummel T, Ural A, Draf W, Bockmuhl U (2008). Predictors of the outcome of nasal surgery in terms of olfactory function. Eur Arch OtoRhinoLaryngol.

[16] Schriever V, Gupta N, Pade J, Szewczynska M, Hummel T (2013). Olfactory function following nasal surgery: a 1-year follow-up. Eur Arch OtoRhinoLaryngology.

[17] Jiang R-S, Lu F-J, Liang K-L, Shiao J-Y, Su M-C, Hsin C-H (2008). Olfactory function in patients with chronic rhinosinusitis before and after functional endoscopic sinus surgery. Am J Rhinol.

[18] Jiang R-S, Su M-C, Liang K-L, Shiao J-Y, Hsin C-H, Lu F-J (2009). Preoperative prognostic factors for olfactory change after functional endoscopic sinus surgery. Am J Rhinol Allerg.

[19] Soler ZM, Sauer DA, Mace JC, Smith TL (2010). Ethmoid histopathology does not predict olfactory outcomes after endoscopic sinus surgery. Am J Rhinol Allerg.

[20] Shin SH, Park JY, Sohn JH (1999). Clinical value of olfactory function tests after endoscopic sinus surgery: a short-term result. Am J Rhinol.

[21] Ehnhage A, Olsson P, Kölbeck KG, Skedinger M, Dahlen B, Ålenius M (2009). Functional endoscopic sinus surgery improved asthma symptoms as well as PEFR and olfaction in patients with nasal polyposis. Allergy.

[22] Preeti Kohli et al (2016). Olfactory Outcomes after Endoscopic Sinus Surgery for Chronic Rhinosinusitis. Otolaryngol Head.

[23] Alt JA, Mace JC, Buniel MCF, Solar ZM, Smith TL (2014). Predictors of Olfactory Dysfunction in Rhinosinusitis Using the Brief Smell Identification Test. Laryngoscope.

[24] Bakhshaee M, Sharifian MR, Ghazizadeh AH, Nahid K, Jalaeian Samani K (2016). Smell Decline as a good Predictor of Sinonasal Polyposis Recurrence after Endoscopic Surgery. Iranian J Otorhinolaryngol.

[25] Bakhshaee M, Jabari F, Ghassemi M, Hourzad SH, Deutscher R, Nahid N (2014). The Prevalence of Allergic Rhinitis in Patients with Chronic Rhinosinusitis. Iran J Otorhinolaryngol.

